# Development of Orally Disintegrating Tablets of Standardized *Rhodiola rosea* Extract

**DOI:** 10.3390/ph18091328

**Published:** 2025-09-04

**Authors:** Oxana Brante, Rihards Talivaldis Bagons, Santa Niedra, Austris Mazurs, Baiba Mauriņa, Jurga Bernatoniene, Konstantins Logviss

**Affiliations:** 1 Laboratory of Finished Dosage Forms, Faculty of Pharmacy, Riga Stradins University, LV-1007 Riga, Latviakonstantins.logviss@rsu.lv (K.L.); 2 Baltic Biomaterials Centre of Excellence, Headquarters at Riga Technical University, LV-1048 Riga, Latvia; baiba.maurina@rsu.lv; 3 Department of Applied Pharmacy, Faculty of Pharmacy, Riga Stradins University, LV-1007 Riga, Latvia; 4 Institute of Pharmaceutical Technologies, Lithuanian University of Health Sciences, LT-50161 Kaunas, Lithuania; 5 Department of Drug Technology and Social Pharmacy, Faculty of Pharmacy, Lithuanian University of Health Sciences, LT-50161 Kaunas, Lithuania

**Keywords:** orally disintegrating tablets, ODT, direct compression, root and rhizome herbal (plant) extract, *Rhodiola rosea*

## Abstract

**Background/Objectives:** *Rhodiola rosea* L. (*Crassulaceae*), a perennial adaptogenic herb native to Northern Europe, Asia, and North America, is renowned for its therapeutic properties attributed to phenolic compounds including flavonoids, phenylethanoids, phenylpropanoids, and cinnamyl alcohol glycosides. The plant’s antioxidant and anti-inflammatory activities align with its traditional use in boosting physical and cognitive performance, reducing fatigue, and improving stress resilience. However, conventional dosage forms present compliance challenges, particularly for vulnerable populations with swallowing difficulties. This study aimed to develop and optimize orally disintegrating tablets (ODTs) containing standardized *Rhodiola rosea* root and rhizome (*RR*) dry extract to ensure rapid disintegration and acceptable taste, thereby improving patient compliance. **Methods:** Dried *Rhodiola rosea* root and rhizome (particle size 2–3 mm) were extracted using 70% m/m ethanol using the fractionated maceration methodology. The resulting dry *RR* extract was standardized to 3.0% m/m rosavin content by blending batches of the extract and analyzed using validated chromatographic methods. The standardized dry extract was formulated into ODTs via direct compression technology. Various excipients were evaluated to achieve rapid disintegration while masking the characteristic bitter taste of *RR* extract. **Results:** The optimized ODT formulation (500 mg, 11 mm ø, 20% standardized *RR* dry extract) disintegrated within 3 min and effectively masking the characteristic bitterness of the *RR* extract. The formulation maintained content uniformity and did not exhibit loss of active compounds during processing, meeting European Pharmacopoeia requirements for ODTs. **Conclusions:** The developed ODTs containing standardized *Rhodiola rosea* extract offer a patient-friendly alternative for oro-mucosal administration, supporting improved compliance in populations with swallowing difficulties while retaining the extract’s phytochemical integrity and sensory acceptability.

## 1. Introduction

### 1.1. Stress and Adaptogens

In today’s fast-paced world, many individuals grapple with a range of psychiatric conditions, including depression, anxiety, and insomnia [1]. There is growing evidence that a potential link exists between these disorders and imbalances in oxidative stress and the body’s antioxidant defense system [2]. Some plants have proven their effectiveness by having a strengthening and calming effect on the human body. Such plants are called “adaptogenic” plants. The European Medicines Agency (EMA), among other sources, notes that adaptogens are pharmacologically active compounds or plant extracts from different plant classes that are believed to enhance the body’s nonspecific resistance to physical, chemical, and biological stressors. They are characterized by low toxicity, broad-spectrum protective effects, and the ability to help normalize physiological functions and support the body during stress. Key examples of adaptogenic plants include *Eleutherococcus senticosus*, *Panax ginseng*, *Schisandra chinensis*, and *Rhodiola rosea*. Adaptogens are reported to reduce stress reactions, prevent exhaustion, and promote recovery from fatigue, although their mechanisms of action are not fully understood and require further clinical validation [3,4]. They have the ability to enhance the body’s stability against physical loads without increasing oxygen consumption. The use of plant adaptogens has a rich history. They have been used by people for hundreds of years in different parts of the world [5]. The most substantial effect of adaptogens is observed in individuals with low or moderate resistance to extreme conditions, and it is almost absent in those with high resistance [6].

### 1.2. Rhodiola rosea as Plant Raw Material

*Rhodiola rosea* L. (commonly named golden root, roseroot), a perennial herbaceous plant from the family *Crassulaceae*, is native to Northern Europe, Asia, and North America, primarily found in rocky, mountainous habitats. It is a well-known adaptogen with biological activity primarily attributed to phenolic compounds—flavonoids, catechins, procyanidins, phenylpropanoids, gallotannins, ellagotannins, and anthocyanins—including various glycosides of these compounds. They possess antioxidant and anti-inflammatory activity and may be responsible for the fatigue-reducing and mental- and physical-performance-enhancing effects attributed to the plant [7,8,9]. Specifically, the phenylethanol derivative salidroside is often associated with adaptogenic activity [10]. Multiple human trials have shown that the use of *R. rosea* can increase work capacity and endurance. According to the EMA/HMPC’s ongoing work and conclusions, arctic root medicines are recognized for their traditional use in providing temporary relief from stress-related fatigue; however, the supporting evidence remains limited, and these findings should serve as a reference for national licensing decisions [11]. The intake of *R. rosea* appears to improve performance during exercise, as well as prolong time to exhaustion during intense work [2,6,12]. In addition, pre-clinical studies found that it can improve the stress response in animals. The exact mechanisms of action are not definitively known. Still, they may include modulation of biochemical processes involved in energy metabolism, as well as direct effects on neurotransmitter release in the central nervous system, as indicated by rodent studies [1,5].

Biologically active substances that have a beneficial effect on the human body accumulate in the underground parts of *R. rosea*, namely in the roots and rhizomes. The dried roots and rhizomes of *R. rosea* contain about 140 different chemical compounds that have been isolated from them, including substances from six large phytochemical groups:

- Phenylpropanoids—rosavin;
- Phenylethanoid derivatives—salidroside and tyrosol;
- Flavonoids—tricine and rodionine;
- Monoterpene derivatives—rosidrol;
- Triterpenes—daucosterol;
- Phenolic acids—gallic acid [1,10,13,14,15].

The main biologically active substances which provide the pharmacological activity of *RR* are considered to be salidroside, a tyrosol glycoside, and rosavin, a cinnamic acid glycoside, which also includes rosin and rosarin [1,10,16,17].

Various methods are used to obtain *RR* extract, with maceration being one of the most widely used [16]. Extractions can be carried out using a variety of solvents, including water, methanol, ethanol, or other organic solvents. The process can be performed under different conditions, such as room temperature or elevated temperature, or using auxiliary methods, such as microwaves, pressure (elevated), or ultrasound [15,18,19]. Both *RR* (as dried vegetable product) and the extracts obtained from this plant material exhibit a taste profile characterized by bitterness and astringency, often perceived as unpleasant. This sensory property poses a significant challenge in the development of palatable oral dosage forms. It is the primary reason for the frequent use of taste-masking strategies in product formulation. It is crucial to find an optimal dose of extracts that is both effective and acceptable to users in terms of taste and that does not exceed toxic doses. Regarding *R. rosea*, the search results do not provide specific toxicity data for *RR* extracts. Generally, *RR* extract is considered safe at typical dosages used in dietary supplements, which range from 200 mg to 600 mg per day. Toxicity may occur at significantly higher doses [10,20].

### 1.3. Orally Disintegrating Tablets (ODTs)

The most common dosage form for plant extracts is hard gelatin capsules; however, this conventional form has the disadvantage of difficulty with swallowing. A more advanced dosage form would be orally disintegrating tablets (ODTs). ODTs are proven to be beneficial to patient compliance, especially within the geriatric, pediatric, or institutionalized patient populations, which have a high incidence of dysphagia. The development of pediatric-friendly ODT formulations has been particularly emphasized for natural extracts, as these formulations can mask unpleasant tastes while providing appropriate dosing for children [21]. Successful patient compliance can also be achieved for patients with psychiatric disorders [22,23]. Additionally, crushing tablets is an inefficient procedure that can lead to loss of drug and reduced dose ingested [24]. The ODT administration method has numerous benefits, including its rapid disintegration, which for some drug substances may help avoid first-pass metabolism, reduce drug degradation, and minimize gastrointestinal breakdown [22,23]. ODTs dissolve quickly in the mouth without the need for water, making them convenient for on-the-go use, especially beneficial for active individuals, travelers, and those with busy lifestyles. The rapid disintegration of ODTs may facilitate partial absorption of active compounds through the oral mucosal membrane, which could potentially lead to a quicker onset of effects. ODTs are designed to break down or dissolve quickly when in contact with saliva. Two primary criteria define an ODT and are key components of quality target product profiles (QTPPs): a disintegration time of 30 s or less and a tablet weight of 500 mg or lower (FDA Guidance for Industry) [22]. However, the Ph. Eur. limit for disintegration time is within 3 min [23]. The administration of drugs through the oro-mucosal route offers the option for both local and systemic delivery. Liquefaction of the ODT occurs on the tongue, followed by the patient swallowing the liquid. Drug substance release from an ODT involves a sequence of processes: initial tablet disintegration, followed by drug dissolution and subsequent absorption, which usually occurs mainly in the intestines. Additionally, unlike effervescent tablets, ODTs do not require the patient to take an extra step of dissolving them in a glass of water before administration [24].

This method of easy and safe administration, combined with the rapid onset of action of the active ingredient, can provide increased bioavailability, better tolerability, and improved patient compliance compared to other conventional solid dosage forms [24,25,26,27]. However, an important aspect is taste and taste masking, which often limits the development of ODTs. Various methods can be used, but the first and simplest one that is worth trying is the addition of a sweetener. If this does not work, more sophisticated methods can be used, which are often more expensive, such as the use of polymers and cyclodextrins [28,29].

### 1.4. Commercial Rhodiola rosea Products

*Rhodiola rosea* root and rhizome (*RR*) is commercially available in various pharmaceutical and nutraceutical forms worldwide. Based on search results of commercial products available on the market, hard gelatin capsules appear to be the predominant dosage form (usually containing 200–400 mg of standardized dry extract), followed by film-coated tablets, with liquid preparations, soft capsules, and powder sachets being less common. Brinckmann et al. (2021) found that the global demand for *R. rosea* ingredients and products has been increasing recently [30,31]. Despite the therapeutic potential of *R. rosea*, the pronounced bitter and astringent taste of the extract limits patient acceptance and compliance. *RR* extract’s primary indication for stress and fatigue relief aligns with patient populations who would benefit from ODT convenience (busy professionals, elderly, weakened patients). We have not found commercially available *RR*-containing ODTs; therefore, our pilot study addresses an identified gap in the market by developing ODTs containing a standardized *RR* dry extract.

## 2. Results

### 2.1. Herbal Drug Dry Extract

The raw material of *Rhodiola rosea* used in this study was tested, and the content of *RR* markers was 0.7 ± 0.003% m/m (*n* = 4) of salidroside and 1.1 ± 0.01% m/m (*n* = 4) of rosavin, which agrees with the minimum contents given in the Ph. Eur. monograph 07/2024: 2893 Rhodiola root and rhizome (min. 0.1% salidroside, min. 0.5% total rosavin) [32].

The main independent extraction variables influencing the quality of the extract considered in our preliminary studies were the degree of grinding of the plant product, the concentration of ethanol used as the extraction solvent, and the drug solvent ratio in each of the two stages of the extraction carried out by a repeated maceration process. The results showed that more efficient extraction, with higher amounts of rosavin (4.699 ± 0.007% m/m, *n* = 4) and salidroside (1.950 ± 0.001% m/m, *n* = 4), and optimal yield can be obtained by using *RR* grinded to 2–3 mm and 70% m/m ethanol as the extraction solvent, in a fractionated two-stage maceration process consisting of 6 h (no stirring) extraction at a drug/solvent ratio of 1:7 m/m, followed by 3 h (6 shaking) extraction at a drug/solvent ratio of 1:5 m/m. This finding aligns with recent advances in *RR* extraction, where researchers have demonstrated that 70–75% *v*/*v* ethanol concentrations provide superior extraction efficiency compared to other solvent systems [17]. Recent studies by *Tsvetov* et al. (2020) explored natural deep eutectic solvents (NADESs) for *RR* extraction, achieving rosavin concentrations of approximately 1000 μg/mL while confirming that aqueous ethanol remains the most practical and efficient solvent for industrial applications [33]. Regarding the influence of the particle size of the plant product subjected to extraction, it was observed that particles smaller than 2 mm tended to compact during extraction, thereby hindering effective solvent penetration and reducing extraction efficiency. Conversely, particles exceeding 3 mm in size provided insufficient surface area for adequate extraction, resulting in suboptimal yields. This finding is consistent with recent particle size optimization studies showing that while generally reducing the particle size increases extraction efficiency by improving surface area contact with solvents, excessive size reduction can lead to bed-blocking and channeling effects [34,35]. Thus, a particle size range of 2–3 mm was identified as optimal for maximizing both the extract quantity and standardization. Good extraction results using methanol as the extractant are reported in the literature [16,18]. However, use of ethanol was preferred due to its superior safety and comparable efficiency relative to methanol [36]. Ethanol with a concentration of 70% m/m gave better extraction results for rosavin and salidroside, while 40% m/m ethanol lagged only slightly in terms of efficiency.

**Figure 1:**
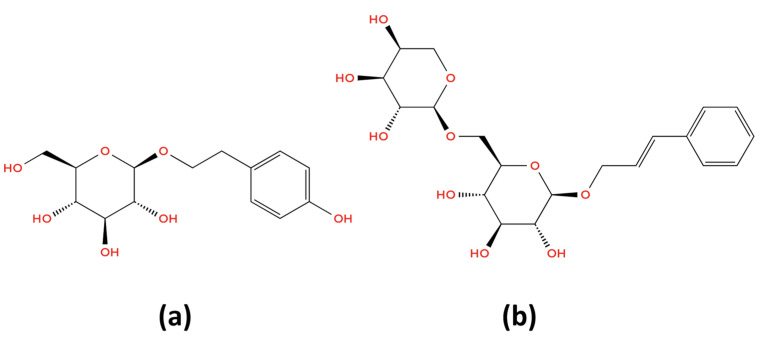
Chemical structure of the two constituents (as *RR* active markers) of interest in quantitative analyses of *RR* dry extract (*DERgenuin* 3:1)-based products. (**a**) Salidroside: 2-(4-Hydroxyphenyl)ethyl β-D-glucopyranoside; (**b**) rosavin: (2E)-3-Phenylprop-2-en-1-yl 6-O-α-L-arabinopyranosyl-β-D-glucopyranoside.

In order to obtain a solid *RR* extract, the hydroalcoholic extract (miscella) resulting from the *RR* extraction (by double maceration) process was further subjected to the process of removing all solvents (by evaporation and then drying), finally resulting in a genuine (native) dry (solid) *RR* extract corresponding to a drug extract ratio (*DER*) of 3:1 (1 g of dry extract being obtained from 3 g of plant product). In order to use it as a drug ingredient in ODTs, the *RR* dry extract (*DERgenuin* 3:1) was standardized to a rosavin content of 3% m/m (by blending batches of the extract, without adding any inert excipient) and then dried until its moisture content reached 4.5% m/m. This herbal extractive dried drug ingredient is designed to contain two quantitatively analyzable constituents (considered as *RR* active markers): salidroside and rosavin (Figure 1).

**Figure 2:**
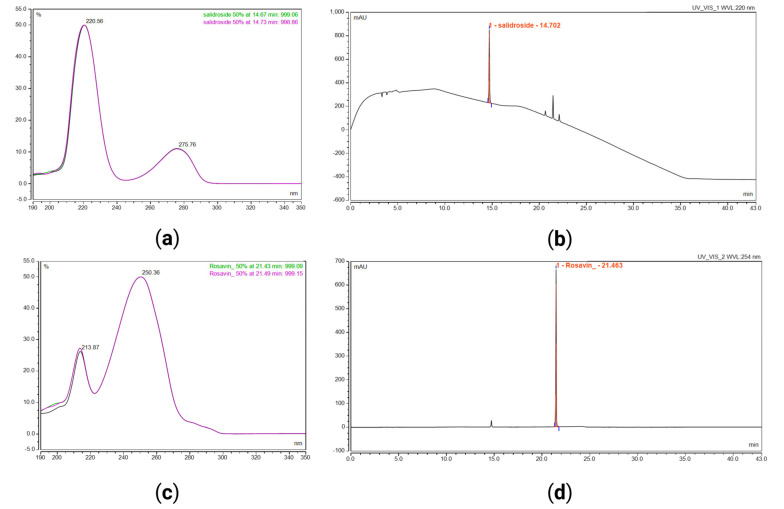
Representative HPLC-UV chromatograms of the reference standard calibration mix and corresponding UV spectra: (**a**) UV spectrum of salidroside, range 190–350 nm; (**b**) representative chromatogram of salidroside standard at 220 nm, concentration 0.53 mg/mL; (**c**) UV spectrum of rosavin, range 190–350 nm; (**d**) chromatogram of rosavin standard at 254 nm, concentration 0.25 mg/mL.

For quantitative analyses of the two *RR* markers (salidroside and rosavin), a HPLC-UV analytical method was developed based on previous studies [37]. The method was validated, including tests of specificity, range, accuracy, and repeatability. The wavelengths for UV detection were selected based on the *RR* markers’ absorption properties and therefore set to 220 nm for salidroside and 254 nm for rosavin, see Figure 2. The method had sufficient selectivity and chromatographic separation of *RR* markers regarding resolution, symmetry, and retention factors [38]. Typical chromatograms of reference standards are shown in Figure 2. The specificity of the stability-indicating method was tested by exposing the *RR* dry extract to acidic (0.1 M HCl, 24 h), basic (0.1 M NaOH, 24 h), oxidative (3% *v*/*v* H_2_O_2_, 24 h), and heat (70 °C, 24 h) conditions. The criteria for resolution ≥ 1.5 and peak purity match factor ≥ 950 were met for all tests. The method was linear within the range of 0.0026–0.63 mg/mL (R = 0.99998; eight-point calibration, y = k × x + m) for salidroside and 0.00025–0.30 mg/mL (R = 0.99998; nine-point calibration, y = k × x + m) for rosavin determination. The limit of quantification (LOQ) was determined using signal-to-noise-based calculation at the lowest calibration concentration. The method was repeatable and accurate. The validated method was found fit for the purpose of determining the content of *RR* markers (rosavin and salidroside) and, therefore, useful for content determination in ODT development. A summary of the analytical method validation is given in Table 1.

**Figure 3:**
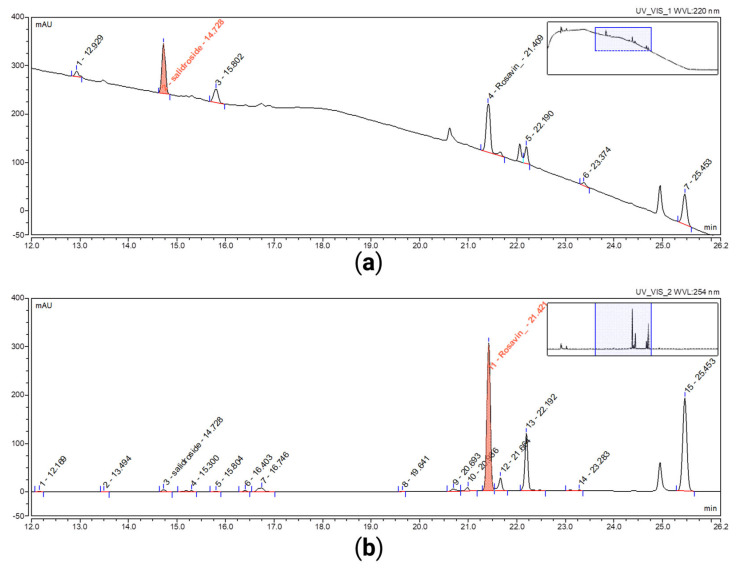
Representative HPLC-UV chromatograms of *RR* dry extract (*DERgenuin* 3:1). (**a**) A chromatogram of standardized *RR* extract at 220 nm, zoomed 12–26 min; (**b**) a chromatogram of standardized *RR* extract at 254 nm, zoomed 12–26 min.

For quantitative analyses of RR extracts, quantification was conducted by eight-point ex-ternal calibration using reference standards and the method described above. The RR dry extract was standardized to contain 3.0% m/m rosavin content by blending batches of the extract. The standardized RR dry extract contained 30.2 ± 0.1 (*n* = 4) mg/g of rosavin and 17.0 ± 0.01 mg/g (*n* = 4) of salidroside. Typical chromatograms of the standardized RR dry extract are shown in Figure 3.

### 2.2. Development of ODT Formulation and Evaluation of Taste-Masking Capacity

The initial formulations were developed based on compositions reported in the scientific literature to establish a technological process for fast-dissolving ODTs [25]. Furthermore, the initial formulations were designed to assess the influence of croscarmellose sodium on tablet disintegration and to evaluate the impact of the selected sweeteners, or their combinations, on the organoleptic properties of the tablets. Thus, twenty OTD formulations (R1–R20) were developed and tested (Table 2) in order to select an optimal composition from the point of view of disaggregation and acceptability of the perceived taste (tested in vivo on six volunteers instructed to rate the intensity of bitterness and astringency on a 12-point scale: 1 = extremely unpleasantly intense; 12 = fully masked). Mannitol (R1), dextrose (R2), and 1:1 combination of both (R3) were initially selected as sweeteners for the formulation. Sensory evaluation revealed that mannitol alone was insufficient to adequately mask the unpleasant taste of the *RR* dry extract (taste score = 3). Consequently, four formulations (R4–R7) were prepared using 3% croscarmellose sodium as the disintegrant and either dextrose or a dextrose–mannitol mixture as sweeteners. The choice of croscarmellose sodium as the primary disintegrant is supported by recent studies on ODTs, which consistently demonstrate its superior performance compared to other superdisintegrants at optimal concentrations. The studies confirm that croscarmellose sodium provides the best balance between rapid disintegration and tablet hardness for a variety of drug substances [39,40]. The influence of citric acid on organoleptic properties (R8, R9), as well as the impact of increasing the *RR* dry extract content from 20% m/m to 30% m/m (R7, R10, R11) on taste acceptability, was subsequently investigated. It was observed that a higher extract concentration (30%), irrespective of the sweetener used, resulted in a marked deterioration in organoleptic properties due to the pronounced astringency of the *RR* dry extract (taste score = 1 for R7 and 2 for both R10 and R11). Therefore, it was decided that the 20% m/m *RR* dry extract would be used in the formulation (Table 2) for further ODT development (R12–R20). It should be emphasized that we attempted higher extract loadings (30% m/m per tablet) for these formulations as well, but all of them exhibited unacceptable bitterness. Thus, we could state that 20% m/m of *RR* dry extract represents the upper limit of feasibility for the chosen excipient system to obtain ODTs by the direct compression method. From a pharmaceutical manufacturer’s perspective, direct compression is the simplest, fastest, and most affordable way to make tablets. Alternative strategies such as granulation or coating the extract in future work may allow increasing the extract load per tablet through advanced formulation techniques.

Although the addition of citric acid (1% in R8, R9, R13, and R15 and 2% in R12, R14, and R16–R20) to formulations containing 20.0% m/m *RR* dry extract led to some improvement in palatability, the effect was insufficient to achieve acceptable taste masking. Furthermore, formulations containing the dextrose–mannitol mixture (as sweeteners) with 3% croscarmellose sodium (as the disintegrant) consistently received lower taste scores in the presence of citric acid (taste enhancer) both at 1% (5 for R9) and 2% (7 for R12), compared to those containing dextrose alone (6 for R8, 7 for R12). This prompted further investigation into the effect of increased citric acid concentration (from 1% to 2%) in formulations using dextrose as the sole sweetener (R12–R16). A significant enhancement in organoleptic properties was observed with the higher level of 2% citric acid but only when reducing the concentration of the disintegrant (from 3% to 1% croscarmellose sodium): R14 (taste score = 8) compared to R13 (taste score = 6). In contrast, increasing the amount of disintegrant (to 5% croscarmellose sodium) produces a significant reduction in organoleptic properties, irrespective of the taste enhancer amount (1% or 2% citric acid): both R15 and R16 had a taste score of 4. So, this and, additionally, the concentration of croscarmellose sodium were found to influence not only the tablet disintegration time and hardness but also taste perception. Tablets containing 1.0% m/m croscarmellose exhibited slower disintegration, while those with 5.0% m/m disintegrated faster but with an intensified unpleasant taste. Accordingly, the croscarmellose concentration was optimized to 3.0% m/m, providing rapid and uniform disintegration without adversely affecting the taste. To further improve taste masking, aspartame (an artificial sweetener) was added to the formulation (R17–R20), varying its concentration in a range of 7.2–7.5%, thus achieving the maximum taste-masking capacity (score 9–10) that is not influenced by the presence of the taste enhancer (citric acid 2%) but only by the concentration of the disintegrant: croscarmellose sodium 3% was determined to have a taste score of 10 (R18, R20), while 2% had a taste score of 9 (R17, R19). The effects of varying the croscarmellose sodium concentration (2–3%) as well as the influence of adding 2% talc (as a lubricant and glidant, favoring compression) on the physical characteristics of the tablets were also evaluated for pre-selected R12-R20 formulations (Table 3). Regarding these formulations, the entire panel of experts who participated in performing the palatability test independently found that the R14 formula provided adequate taste masking for initial development purposes and concluded that the levels of bitterness and astringency were within acceptable ranges. However, we would like to point out that we were unable to completely mask the taste (grade 10 out of 12 even for aspartame-containing formulations).

The addition of aspartame (R17–R20) significantly improved the organoleptic assessment of the formulations, but powder sticking to the punches and the tablet capping effect were observed. Therefore, of all the tablet formulations, R-14 was selected for further ODT development due to its superior organoleptic and physical properties to proceed from smaller 200 mg ODTs (with content of 40 mg standardized *RR* dry extract) to bigger 500 mg ODTs (with content of 100 mg standardized *RR* dry extract) which better correspond to recommended daily doses and the acceptable number of dosage forms to be taken daily [11]. It should be noted that we opted for a limited, qualitative in-house sensory assessment to obtain preliminary evidence of effective taste masking. To support our approach, we can cite several peer-reviewed research papers that evaluated taste masking using a small group of human volunteers or research team members, without the use of validated electronic tongue (e-tongue) analytical devices or large sensory panels: A study developed taste-masked microparticles for orally disintegrating tablets [41]. The effectiveness of taste masking was evaluated by a panel of six human volunteers. In a recent review article on some excipients designed for pharmaceutical purposes, Adamkiewicz et al. (2023) provides the number of volunteers (six being most frequent) participating in the reviewed works analyzing the taste-masking properties of cyclodextrins [42]. Another review (Yoo O. et al., 2023) on pediatric medicinal formulation development notes that highly trained sensory panels are not always necessary during the developmental phase and that untrained panels, when provided with appropriate reference samples, can yield results comparable to those of trained panels [43]. In this exploratory context, our research goal was to develop proof-of-concept ODTs of *RR* dry extract and to perform early-stage characterization, not to support regulatory filings or market entry, which would require more exhaustive and validated sensory testing methodologies.

Due to the limited quantity of the extract, the flow characteristics of the powder blend were determined only for the formulation selected for taste quality, which was prepared in an amount of 200 g. The flow through an orifice failed to provide results, as the powder did not flow through either the 10 mm or 15 mm orifice. Therefore, to evaluate the powder blend’s flow properties, we used only the angle of repose measurement, calculation of the compressibility index, and determination of the Hausner ratio. The angle of repose (37°), Hausner ratio (1.21), and compressibility index (Carr’s index) (17.5%) of the powder mass exhibited fair-to-passable powder flow characteristics (Table 4). These values align with current pharmaceutical powder technology standards and recent ODT formulation guidelines. All three methods agree that the powder blend exhibits “fair” flowability—according to pharmacopoeial standards, this means the material is likely suitable for many manufacturing processes but is not an ideal, free-flowing powder. Some attention to flow improvement could be considered if formulation or manufacturing experiences issues. According to the literature, Hausner ratios between 1.15 and 1.25 indicate acceptable flow properties for direct compression applications, while Carr’s index values of 15–20% represent fair flow characteristics suitable for tablet manufacturing [44]. No segregation was observed during tapped density measurements of the powder blend.

The R14 powder blend, selected as the optimal composition, was used to prepare a second series of ODTs (mass of 500 mg, 11 mm ø) at a compression pressure of 150 MPa; the resulting tablets showed mass uniformity (as the mass variation was no more than 5%) and an average resistance to breaking (hardness test) of 73 N, were resistant to friction (friability of 0.17%, so below the 1.0% set as the acceptance criterion according to Ph. Eur.), and disintegrated in water in an average of 2 min. (under 3 min. according to the Ph. Eur. tablet monograph on ODTs section).

The content of the two *RR* active markers after the preparation of the tablets was determined to be 0.61% rosavin and 0.31% salidroside (Table 5), which corresponds to the amount of the standardized *RR* dry extract (*DERgenuin* 3:1) in the formulation, meaning there is no loss of the active compounds during tablet preparation. After 6 months of preservation at 25 °C, the content of *RR* active markers remained constant (Table 5), without significant changes, with the content variances in the tablet being determined within the error of determination (with relative standard deviation—RSD—of 5%).

## 3. Materials and Methods

### 3.1. Chemicals and Reagents

The *Rhodiola rosea* dry plant (roots), originating from the Altai region and harvested in 2023, used in this study as the plant material (*RR*) was kindly donated by Elpis Ltd. (Riga, Latvia). Mannitol (Pearlitol 200SD, Roquette, Lestrem, France), dextrose, and aspartame were kindly donated by Grindeks JSC (Riga, Latvia). Croscarmellose sodium was purchased from Farmalabor (Milan, Italy); citric acid was purchased from Orkla Ltd. (Riga, Latvia); talc and magnesium stearate were purchased from Fanex Ltd. (Riga, Latvia); ethanol 96.3% (which served to prepare in the laboratory, by dilution with purified water, ethanol 70% m/m used as an extraction solvent) was purchased from Aroma Floris Ltd. (Riga, Latvia). All chemicals used for chromatographic analysis were HPLC-grade or higher. Acetonitrile (≥99.9%, Fisher Scientific, Loughborough, UK), ammonium acetate (LC-MS, Fisher Scientific, Fair, Lawn, NJ, USA), and methanol (≥99.9%, CHROMASOLV, Seelze, Germany); analytical standards for quantification of salidroside (≥99%, Sigma-Aldrich, Saint Louis, MO, USA) and rosavin (≥99%, Sigma-Aldrich, Saint Louis, MO, USA) were used. Ultrapure water was prepared using the Stakpure purification system (OmniaTap 6, Niederahr, Germany). Hydrochloric acid (37%, pure, pharma-grade, Honeywell, Seelze, Austria), sodium hydroxide (≥98%, Sigma-Aldrich, Darmstadt, Germany), and hydrogen peroxide (≥30%, Sigma-Aldrich, Saint Louis, CA, USA) were used for the degradation study.

### 3.2. Standardized RR Dry Extract Used as Drug (Active Ingredient) in ODTs

A genuine (native) *RR* dry extract was produced according to Ph. Eur. requirements for dry extracts (*extracta sicca*) [45] using the following methodology: The *RR* plant material (consisting of dried roots and rhizomes) was milled to 2–3 mm particle size using a knife mill (Retsch SM300, Haan, Germany) with a 6 mm sieve and then sieved through analytical sieves (Retsch, Haan, Germany) of different sizes and subjected to fractionated maceration using ethanol 70% m/m as an extraction solvent. The extraction process consisted of a first extraction with a drug/solvent ratio of 1:7 (m/m) and an extraction time of 6 h without stirring and a second extraction with a drug/solvent ratio of 1:5 (m/m) and an extraction time of 3 h with shaking every 30 min. The resulting miscella (extraction liquor) was combined, concentrated using a rotary evaporator (Strike 380 HomTron Wiggens GmbH, Wuppertal, Germany), and dried in a vacuum oven (OV4–30 Jeiotech Co., Ltd., Daejeon, Republic of Korea) to obtain the dry extract. The dry extract was standardized to 3.0% rosavin content by blending batches of the extract according to Ph. Eur. requirements for standardized extracts. The extract mass was dried until its moisture content was less than 5%. The moisture of the extract was measured with a moisture analyzer (HX204 Mettler Toledo AG, Greifensee, Switzerland) according to the Ph. Eur. 2.2.32. Loss on Drying monograph [46]. No inert excipients were added during standardization. The standardized dry extract was stored in well-closed containers, protected from light and moisture, at a temperature not exceeding 25 °C.

The Rhodiola rosea root and rhizome (RR) dry extract prepared and used in this study is classified as a standardized extract according to the European Pharmacopoeia (Ph. Eur.) monograph Herbal Drug Extracts definition and the EMA Guideline on declaration of herbal substances and herbal preparations in herbal medicinal products [45,47], as it is adjusted to a defined content of rosavin (3.0%), a constituent with known therapeutic activity.

### 3.3. Powder Mixture Intended for Compression

The standardized dry extract of *Rhodiola rosea* root and rhizome (*RR*) containing at least 3% (30 mg/g) rosavin and 1% (10 mg/g) salidroside was ground in a mortar and sieved through an analytical sieve (Retsch, Haan, Germany) to ensure a particle size of less than 1 mm.

Out of the twenty developed and tested formulations, one was ultimately selected based on its optimal performance. Based on data from the studied literature [25,48] and through preliminary laboratory tests, 20 mixtures (R1–R20) intended for direct compression in the form of ODTs were formulated, according to the percentage quantities presented in Table 2. During the formulation preparation, all raw materials were accurately weighed and then sequentially introduced into a dedicated mixing vessel. Initially, mannitol and/or dextrose were incorporated, followed by the addition of the standardized *RR* dry extract; then, aspartame, citric acid, croscarmellose sodium, and talc were added in the quantities indicated in Table 2. Magnesium stearate was introduced last, functioning as a lubricant to enhance powder flow and prevent adhesion. Ingredient blending and lubrication were performed using a double-cone blender (DVC Developer Comasa, Barcelona, Spain).

The angle of repose of the powder mass of the R14 formulation (*n* = 3) was determined with a flowability tester (GTB2 Erweka GmbH, Langen, Germany). The tapped density, Hausner ratio, and compressibility index (Carr’s index) were determined using the tapped density tester (SVM II Erweka GmbH, Langen, Germany) according to the Ph. Eur. monographs 2.9.34. Bulk Density of Powders and 2.9.36. Powder Flow [49,50]. The results are presented in Table 4.

### 3.4. Tableting and Testing of ODTs with Content of Standardized RR Dry Extract

The tableting of all formulations was performed by the direct compression method using a benchtop compaction simulator (STYL One Nano, Medelpharm, Beynost, France). The punches used were Euro B format, curved on both sides, with a diameter (ø) of 7 mm (for all R1–R20 formulations) and 11 mm (for the selected 500 mg R14 formulation). Tableting was performed using a V-shaped compression profile, a punch speed configuration of 10.0 mm/s, and tableting pressure in the range of 100–150 MPa.

Tablet hardness (N), thickness, and weight were determined using a fully automated tablet tester (ST50 WTDH Sotax AG, Aesch, Switzerland) for 7 mm tablets R12–R20 (*n* = 3) and for 11 mm tablets R14 (*n* = 10) [51,52]. The disintegration time for finished tablets (*n* = 3) was measured using a disintegration tester (ZT 732, Erweka GmbH, Langen, Germany), Test A without discs, according to Ph. Eur. monograph 2.9.1. Disintegration time [53]. Friability was determined using a friability tester (FRV 100i, Copley Scientific Ltd., Nottingham, UK) for 13 tablets (6.5 g) according to Ph. Eur. monograph 2.9.7. Friability of uncoated tablets [54]. The results are presented in Table 4.

Stability testing was conducted over 6 months under room storage conditions (25 °C, 60% RH) in a constant-climate chamber (HPP1060eco, Memmert GmbH + Co. KG, Büchenbach, Germany). The tablets were analyzed chromatographically for rosavin and salidroside content immediately after tableting and after 6 months of storage in the stability chamber at room temperature (25 °C). The results are presented in Table 5.

### 3.5. Evaluation of Taste Acceptability (Palatability Test)

The taste characteristics of the ODTs were evaluated by a small internal panel of experts. The panel consisted of six evaluators (three females, three males) aged 23 to 50 years, all of whom had previous experience in evaluating pharmaceutical dosage forms. Each evaluator independently tasted each of the R1–R20 ODTs with content of standardized *RR* dry extract (2 tablets of each formulation in randomized order) and rated the intensity of its characteristic bitterness and astringency on a 12-point scale (1 = extremely unpleasantly intense, 2 = extremely unpleasant, 3 = very unpleasant, 4 = unpleasant, 5 = moderately unpleasant, 6 = slightly unpleasant, residual bitterness/astringency, 7 = astringency noticeable, 8 = astringency noticeable, but overall acceptable, 9 = slightly noticeable astringency aftertaste, 10 = no unpleasant taste, 11 = taste pleasant, fully acceptable, and 12 = taste completely pleasant, no bitterness/astringency present). The evaluation was conducted under controlled laboratory conditions, with panelists rinsing their mouths with water before and between samples. There was an interval of no less than 10 min between tasting tablets, to avoid taste overload.

### 3.6. HPLC-UV Analytical Method

Salidroside and rosavin are the two active markers in the *RR* extract and tablets considered for quantification. For this purpose, high-performance liquid chromatography with ultraviolet detection (HPLC-UV) was used. A HPLC system, Vanquish CORE chromatograph (Thermo Scientific, Germering, Germany), equipped with a quaternary pump and a diode array detector was used. Chromatographic separation was achieved using an XBridge BEH Phenyl column, 150 × 4.6 mm, particle size of 2.5 µm, and pore size of 145 Å (Waters, Milford, CT, USA). The chromatographic method conditions were as follows: gradient elution consisting of mobile phase A, 100 mM ammonium acetate aqueous solution, and mobile phase B, acetonitrile, at flow rate 0.5 mL/min, starting with 1% of eluent B for 3 min equilibration time, 2–10 min ramp to 85% eluent B, maintained for 2 min, and 12–31 min 25 % eluent B; 31–40 min 1% eluent B run till 43 min. The sampler temperature was set to 10 °C, and the sample injection volume was 2.00 µL. The column compartment was maintained at 30 °C, and the wavelengths for UV detection were 220 nm for salidroside and 254 nm for rosavin. Chromatographic data system Chromeleon Ver. 7.3.1. (6535) (Thermo Fisher Scientific, Waltham, MA, USA) was used for chromatographic data processing. The in-house analytical method was validated at the fit-for-purpose level considering the ICH Q2 (R2) Guideline on the validation of analytical procedures [55].

Each sample of the *RR* dry extract was prepared in duplicate by accurately weighing approximately 125 mg dry extract into a 25 mL volumetric flask, dissolving, and diluting up to volume with a diluent (50:50, methanol–water, *v*/*v*). The sample was filtered through a 0.45 µm PVDF membrane syringe filter (Merck Millipore, Carrigtwohill, Ireland) and injected.

The sample of *RR* plant material was prepared in duplicate according to the sample preparation procedure in the Ph. Eur. monograph 07/2024: 2893 Rhodiola root and rhizome [32] by accurately weighing approximately 250 mg of powdered herbal drug into a 25 mL volumetric flask, dissolving, diluting up to volume with a 75:25 methanol–water *v*/*v* solution, sonicating for 30 min in an ultrasound bath (Bandelin, Berlin, Germany), filtering through a 0.45 µm PVDF membrane syringe filter, and injecting.

Each sample of ODTs with content of the standardized *RR* dry extract was prepared in duplicate by accurately weighing a tablet in a 50 mL volumetric flask, dissolving in diluent, sonicating for 10 min, and filtering through a 0.45 µm PVDF membrane syringe filter.

Samples of reference standard stock solutions were prepared by accurately weighing approximately 20 mg of each reference substance into a separate 10 mL volumetric flask, dissolving, and diluting up to volume with diluent. Calibration standards were prepared by combining reference standard stock solutions and diluting with diluent to concentrations of 0.50, 0.40, 0.25, 0.05, 0.025, and 0.005 mg/mL of salidroside and 0.25, 0.20, 0.12, 0.025, 0.012, and 0.0025 mg/mL of rosavin.

## 4. Conclusions

The development of ODTs containing standardized *Rhodiola rosea root and rhizome* (*RR*) dry extract presented significant challenges in achieving the effective masking of the unpleasant bitter and astringent taste, characteristic of the plant material, while maintaining favorable technological properties. Among the sweeteners evaluated, in the process of the formulation and optimization of the tablet composition, dextrose alone provided superior masking of the *RR* extract’s astringent and unpleasant taste compared to mannitol or their combination. The addition of citric acid further improved palatability, particularly at higher concentrations, but was insufficient to achieve entirely acceptable taste masking. In our study, the maximum amount of standardized *RR* dry extract (3.0% rosavin) in a tablet that allows maintaining an acceptable taste was found to be 20% in a tablet of 200 mg (40 mg/tablet). At this content of *RR* dry extract in the tablet, increasing the concentration of the disintegrant croscarmellose sodium to 5% accelerated tablet disintegration but exacerbated the perception of bitterness. Conversely, lower concentrations slowed disintegration without providing a substantial taste benefit. Thus, a 3% concentration was identified as optimal for balancing the disintegration time and organoleptic properties, but, depending on the sweetener, the acceptability of the taste was maintained even when the concentration was reduced to 1%. Although the incorporation of aspartame markedly enhanced taste masking, it adversely affected powder flow and tablet manufacturability, resulting in sticking and capping during compression. Out of the twenty formulations studied, one (R14), containing 74.5% dextrose as the sweetener and 1% croscarmellose sodium as the disintegrant, was selected for its optimal balance between taste masking, disintegration, and physical characteristics. The powder blend of this composition exhibited acceptable flow properties, and the produced tablets (500 mg) maintained the stability of the active ingredient (100 mg standardized *RR* dry extract, containing 3 mg rosavin) over a six-month period of storage at 25 °C. While the unpleasant taste of the *RR* dry extract could not be masked entirely, the selected formulation achieved a level of palatability considered acceptable for patient use.

## Figures and Tables

**Table 1:** Analytical HPLC-UV method validation results.

Objective	Parameter	Acceptance Criteria	Result, Rosavin	Result, Salidroside	Observations
Specificity	Resolution	Rs ≥ 1.5	2.0	5.8	*n* = 16
	Peak purity match factor	PPMF ≥ 950	984	985	*n* = 16
Selectivity	Symmetry factor	0.8 ≤ A_s_ ≤ 1.8	0.9	0.9	*n* = 74
	Capacity factor	2 ≤ k’ ≤ 10	6	3	*n* = 74
Linearity	Correlation coefficient	R ≥ 0.997	0.99998	0.99998	*n* = 20
	Standard Error	-	0.11	0.13	*n* = 20
	Intercept	-	0.0577	0.0941	*n* = 20
	Slope	-	168.49	88.92	*n* = 20
	Residual plot	No trend	No trend	No trend	*n* = 20
Quantification limit	Limit of quantification	LOQ ≤ 0.01 mg/mL	0.00011 mg/mL	0.0014 mg/mL	*n* = 3
	Signal-to-noise ratio	S/N ≥ 10	24	19	*n* = 3
Repeatability	Area	RSD ≤ 1%	0.1	0.1	*n* = 6
	Retention time	RSD ≤ 1%	0.02	0.04	*n* = 12
	Content	RSD ≤ 5%	0.6	1.7	*n* = 6
Intermediate precision	Retention time	RSD ≤ 1%	0.1	0.2	*n* = 24
	Content	RSD ≤ 5%	3.0	4.3	*n* = 8
Accuracy	Recovery (80% level)	95–105%	96	98	*n* = 3
	Recovery (100% level)	95–105%	97	98	*n* = 3

**Table 2:** Formulations studied as ODTs (200 mg, 7 mm ø) with content of standardized *RR* dry extract (*DERgenuin* 3:1, 3.0% rosavin) and palatability test results.

ODT	Formulation Variables (Ingredient %)								Taste Evaluation (Score 1–12)
	Herbal Drug Extract	Sweetener, Flavor Enhancer				Disintegrant	Glidant, Lubricant		
		Dextrose	Mannitol	Aspartame	Citric Acid	Croscarmellose Sodium	Talc	Magnesium Stearate	
R-1	20.0	-	77.5	-	-	-	2.0	0.5	3
R-2	20.0	77.5	-	-	-	-	2.0	0.5	3
R-3	20.0	38.8	38.7	-	-	-	2.0	0.5	3
R-4	20.0	-	74.5	-	-	3.0	2.0	0.5	3
R-5	20.0	74.5	-	-	-	3.0	2.0	0.5	4
R-6	20.0	37.3	37.2	-	-	3.0	2.0	0.5	5
R-7	30.0	64.5	-	-	-	3.0	2.0	0.5	1
R-8	20.0	73.5	-	-	1.0	3.0	2.0	0.5	6
R-9	20.0	36.8	36.7	-	1.0	3.0	2.0	0.5	5
R-10	30.0	63.5	-	-	1.0	3.0	2.0	0.5	2
R-11	30.0	31.8	31.7	-	1.0	3.0	2.0	0.5	2
R-12	20.0	72.5	-	-	2.0	3.0	2.0	0.5	7
R-13	20.0	75.5	-	-	1.0	1.0	2.0	0.5	6
R-14	20.0	74.5	-	-	2.0	1.0	2.0	0.5	8
R-15	20.0	71.5	-	-	1.0	5.0	2.0	0.5	4
R-16	20.0	70.5	-	-	2.0	5.0	2.0	0.5	4
R-17	20.0	66.2	-	7.3	2.0	2.0	2.0	0.5	9
R-18	20.0	65.3	-	7.2	2.0	3.0	2.0	0.5	10
R-19	20.0	68.0	-	7.5	2.0	2.0	-	0.5	9
R-20	20.0	67.1	-	7.4	2.0	3.0	-	0.5	10

**Table 3:** Pharmacotechnical characteristics of ODTs (7 mm ø) containing 20% standardized *RR* dry extract (*DERgenuin* 3:1, 3.0% rosavin) with improved taste sensory perception.

Parameter (mean ± SD, *n* = 3)	R-12	R-13	R-14	R-15	R-16	R-17	R-18	R-19	R-20
Disintegration time (s)	17 ± 13.2	36 ± 6.1	44 ± 12.5	15 ± 6.8	32 ± 11.5	24 ± 9.0	43 ± 28.7	20 ± 8.7	110 ± 62.1
Uniformity of mass (mg)	202.2 ± 7.7	201.3 ± 3.8	200.3 ± 4.3	202.7± 5.9	201.8 ± 3.9	201.9 ± 0.7	198.5 ± 8.2	199.4± 5.8	202.4 ± 4.8
Hardness (N)	41.5 ± 10.6	45.5 ± 0.7	43.3 ± 6.8	32.3 ± 0.1	35.3 ± 4.7	39.3 ± 6.7	21.0 ± 10.1	27.0 ± 2.8	36.0 ± 9.8

**Table 4:** Characteristics of the optimal composition (R14): powder blend (20% standardized *RR* dry extract) and ODTs (500 mg, 11 mm ø) prepared by direct compression.

Parameter (Mean ± SD)	Powder Blend (20% Standardized *RR* Dry Extract)	ODTs (100 mg Standardized *RR* Dry Extract) (*n* = 10)
Angle of repose (°) (*n* = 3)	37.0 ± 16.3	-
Hausner ratio (HR)	1.21	-
Carr’s index (CI) (%)	17.5	-
Bulk density (g/mL)	0.60	-
Tapped density (g/mL)	0.73	-
Taste score (1–12 scale)		8
Mass uniformity (mg)	-	499.2 ± 4.69
Tablet thickness (mm) (*n* = 10)	-	5.13 ± 0.018
Tablet hardness (N) (*n* = 10)	-	73 ± 4.8
Friability (%) (*n* = 10)	-	0.169
Disintegration time (s)	-	119.6 ± 19.5
Acceptance value (AV) based on the mass variation	-	11.25

**Table 5:** Optimal composition R14: *RR* active markers determined in ODTs (500 mg, 11 mm ø) prepared by direct compression.

Pharmaceutical Ingredient	Declared mg/tab	Determined *RR* Active Marker Content mg/tab (*Mean ± SD*, *n* = 4)		
		Sample	Rosavin	Salidroside
*RR* dry extract (3% rosavin)	100	Preparation	3.03 ± 0.02	1.57 ± 0.02
Dextrose (sweetener)	372.5			
Croscarmellose sodium (disintegrant)	5			
Citric acid (flavor enhancer)	10	6-month storage	2.95 ± 0.13	1.65 ± 0.08
Talc (glidant, lubricant)	10			
Magnesium stearate (lubricant)	2.5			

## Data Availability

The original contributions presented in the study are included in the article, further inquiries can be directed to the corresponding author.

## Abbreviations

ODTs	Orally disintegrating tablets
*RR*	*Rhodiola rosea* root and rhizome
HPLC	High-performance liquid chromatography
LOQ	Limit of quantification

## References

[1] Ivanova Stojcheva E., Quintela J.C. (2022). The Effectiveness of *Rhodiola rosea* L. Preparations in Alleviating Various Aspects of Life-Stress Symptoms and Stress-Induced Conditions—Encouraging Clinical Evidence. Molecules.

[2] Wal A., Wal P., Rai A.K., Tiwari R., Prajapati S.K., Bagchi D., Nair S., Sen C.K. (2019). Chapter 34—Adaptogens With a Special Emphasis on *Withania somnifera* and *Rhodiola rosea*. Nutrition and Enhanced Sports Performance.

[3] Panossian A., Efferth T. (2022). Network Pharmacology of Adaptogens in the Assessment of Their Pleiotropic Therapeutic Activity. Pharmaceuticals.

[4] EMEA (2008). Reflection Paper on the Adaptogenic Concept.

[5] Panossian A., Hamm R., Wikman G., Efferth T. (2014). Mechanism of action of Rhodiola, salidroside, tyrosol and triandrin in isolated neuroglial cells: An interactive pathway analysis of the downstream effects using RNA microarray data. Phytomedicine.

[6] Tinsley G.M., Jagim A.R., Potter G.D.M., Garner D., Galpin A.J. (2024). *Rhodiola rosea* as an adaptogen to enhance exercise performance: A review of the literature. Br. J. Nutr..

[7] Bawa A.S., Khanum F. (2009). Anti-inflammatory activity of *Rhodiola rosea*—“a second-generation adaptogen”. Phytother. Res..

[8] Jafari M., Juanson Arabit J.G., Courville R., Kiani D., Chaston J.M., Nguyen C.D., Jena N., Liu Z.Y., Tata P., Van Etten R.A. (2022). The impact of *Rhodiola rosea* on biomarkers of diabetes, inflammation, and microbiota in a leptin receptor-knockout mouse model. Sci. Rep..

[9] Liang K., Ma S., Luo K., Wang R., Xiao C., Zhang X., Gao Y., Li M. (2024). Salidroside: An Overview of Its Promising Potential and Diverse Applications. Pharmaceuticals.

[10] Bernatoniene J., Jakstas V., Kopustinskiene D.M. (2023). Phenolic Compounds of *Rhodiola rosea* L. as the Potential Alternative Therapy in the Treatment of Chronic Diseases. Int. J. Mol. Sci..

[11] EMEA (2014). Rhodiolae Roseae Rhizoma Et Radix-Herbal Medicinal Product.

[12] Parisi A., Tranchita E., Duranti G., Ciminelli E., Quaranta F., Ceci R., Cerulli C., Borrione P., Sabatini S. (2010). Effects of chronic *Rhodiola rosea* supplementation on sport performance and antioxidant capacity in trained male: Preliminary results. J. Sports Med. Phys. Fit..

[13] Polumackanycz M., Konieczynski P., Orhan I.E., Abaci N., Viapiana A. (2022). Chemical Composition, Antioxidant and Anti-Enzymatic Activity of Golden Root (*Rhodiola rosea* L.) Commercial Samples. Antioxidants.

[14] Panossian A., Wikman G., Sarris J. (2010). Rosenroot (*Rhodiola rosea*): Traditional use, chemical composition, pharmacology and clinical efficacy. Phytomedicine.

[15] Zakharenko A.M., Razgonova M.P., Pikula K.S., Golokhvast K.S. (2021). Simultaneous Determination of 78 Compounds of *Rhodiola rosea* Extract by Supercritical CO(2)-Extraction and HPLC-ESI-MS/MS Spectrometry. Biochem. Res. Int..

[16] Tolonen A., Pakonen M., Hohtola A., Jalonen J. (2003). Phenylpropanoid glycosides from *Rhodiola rosea*. Chem. Pharm. Bull..

[17] Kosakowska O., Bączek K., Przybył J.L., Pióro-Jabrucka E., Czupa W., Synowiec A., Gniewosz M., Costa R., Mondello L., Węglarz Z. (2018). Antioxidant and Antibacterial Activity of Roseroot (*Rhodiola rosea* L.) Dry Extracts. Molecules.

[18] Iheozor-Ejiofor P., Dey E.S. (2009). Extraction of rosavin from *Rhodiola rosea* root using supercritical carbon dioxide with water. J. Supercrit. Fluids.

[19] Langeder J., Grienke U. (2021). A supercritical fluid workflow for the quality assessment of herbal drugs and commercial preparations from *Rhodiola rosea*. Phytochem. Anal..

[20] Edwards D., Heufelder A., Zimmermann A. (2012). Therapeutic effects and safety of *Rhodiola rosea* extract WS^®^ 1375 in subjects with life-stress symptoms–results of an open-label study. Phytother. Res..

[21] Suksawat T., Brniak W., Łyszczarz E., Wesoły M., Ciosek-Skibińska P., Mendyk A. (2024). Orodispersible Dosage Forms with Rhinacanthin-Rich Extract as a Convenient Formulation Dedicated to Pediatric Patients. Pharmaceuticals.

[22] FDA (2008). Guidance for Industry: Orally Disintegrating Tablets.

[23] EDQM (2025). Tablets. European Pharmacopoeia.

[24] Chinwala M. (2020). Recent Formulation Advances and Therapeutic Usefulness of Orally Disintegrating Tablets (ODTs). Pharmacy.

[25] Ghourichay M.P., Kiaie S.H., Nokhodchi A., Javadzadeh Y. (2021). Formulation and Quality Control of Orally Disintegrating Tablets (ODTs): Recent Advances and Perspectives. BioMed Res. Int..

[26] Ivanovska V., Rademaker C.M.A., van Dijk L., Mantel-Teeuwisse A.K. (2014). Pediatric Drug Formulations: A Review of Challenges and Progress. Pediatrics.

[27] Kelly J., D’Cruz G., Wright D. (2010). Patients with dysphagia: Experiences of taking medication. J. Adv. Nurs..

[28] Ozon E.A., Novac M., Gheorghe D., Musuc A.M., Mitu M.A., Sarbu I., Anuta V., Rusu A., Petrescu S., Atkinson I. (2022). Formation and Physico-Chemical Evaluation of Nifedipine-hydroxypropyl-β-cyclodextrin and Nifedipine-methyl-β-cyclodextrin: The Development of Orodispersible Tablets. Pharmaceuticals.

[29] Sanjay L.R., Ashokbhai M.K., Ghatole S., Roy S., Kashinath K.P., Kaity S. (2024). Strategies for beating the bitter taste of pharmaceutical formulations towards better therapeutic outcomes. RSC Pharm..

[30] Brinckmann J.A., Cunningham A.B., Harter D.E.V. (2021). Running out of time to smell the roseroots: Reviewing threats and trade in wild *Rhodiola rosea* L.. J. Ethnopharmacol..

[31] Kołtun-Jasion M., Czerwiec K., Parzonko A., Bakiera A., Ożarowski M., Kiss A.K. (2025). Comprehensive profiling of *Rhodiola rosea* roots and corresponding products: Phytochemical insights and modulation of neuroinflammation in BV2 microglial cell model. Front. Pharmacol..

[32] EDQM, European Directorate for the Quality of Medicines & HealthCare (2025). Rhodiola root and rhizome. European Pharmacopoeia.

[33] Tsvetov N., Paukshta O., Fokina N., Volodina N., Samarov A. (2023). Application of Natural Deep Eutectic Solvents for Extraction of Bioactive Components from *Rhodiola rosea* (L.). Molecules.

[34] Putra N.R., Rizkiyah D.N., Aziz A.H.A., Mamat H., Jusoh W.M.S.W., Idham Z., Yunus M.A.C., Irianto I. (2023). Influence of particle size in supercritical carbon dioxide extraction of roselle (*Hibiscus sabdariffa*) on bioactive compound recovery, extraction rate, diffusivity, and solubility. Sci. Rep..

[35] Prasedya E.S., Frediansyah A., Martyasari N.W.R., Ilhami B.K., Abidin A.S., Padmi H., Fahrurrozi, Juanssilfero A.B., Widyastuti S., Sunarwidhi A.L. (2021). Effect of particle size on phytochemical composition and antioxidant properties of Sargassum cristaefolium ethanol extract. Sci. Rep..

[36] Wang S., Feng Y., Zheng L., He P., Tan J., Cai J., Wu M., Ye X. (2023). Rosavin: Research Advances in Extraction and Synthesis, Pharmacological Activities and Therapeutic Effects on Diseases of the Characteristic Active Ingredients of *Rhodiola rosea* L.. Molecules.

[37] Ajdert P., Jan L., Burman R. (2022). Liquid chromatographic method for the quantification of salidroside and cinnamyl alcohol glycosides for quality control of golden root (*Rhodiola rosea* L.). J. Appl. Res. Med. Aromat. Plants.

[38] EDQM (2025). Chromatographic Separation Techniques. European Pharmacopoeia.

[39] Desai P.M., Er P.X., Liew C.V., Heng P.W. (2014). Functionality of disintegrants and their mixtures in enabling fast disintegration of tablets by a quality by design approach. AAPS PharmSciTech.

[40] Ali A.T., Nasir F., Hidayatullah T., Pervez S., Rabqa Zainab S., Gohar S., Ur Rahman A., Khattak M.A., Alasmari F., Neau S.H. (2025). Quality by Design Formulation Approach for the Development of Orodispersible Tablets of Dexlansoprazole. Drug Des. Dev. Ther..

[41] Sutthapitaksakul L., Thanawuth K., Dass C.R., Sriamornsak P. (2021). Optimized Taste-Masked Microparticles for Orally Disintegrating Tablets as a Promising Dosage Form for Alzheimer’s Disease Patients. Pharmaceutics.

[42] Adamkiewicz L., Szeleszczuk Ł. (2023). Review of Applications of Cyclodextrins as Taste-Masking Excipients for Pharmaceutical Purposes. Molecules.

[43] Yoo O., von Ungern-Sternberg B.S., Lim L.Y. (2023). Paediatric Medicinal Formulation Development: Utilising Human Taste Panels and Incorporating Their Data into Machine Learning Training. Pharmaceutics.

[44] Maheshwari R., Todke P., Kuche K., Raval N., Tekade R.K., Tekade R.K. (2018). Chapter 17-Micromeritics in Pharmaceutical Product Development. Dosage Form Design Considerations.

[45] EDQM (2025). Herbal Drug Extracts. European Pharmacopoeia.

[46] EDQM (2025). Loss on Drying. European Pharmacopoeia.

[47] EMEA (2010). Guideline on Declaration of Herbal Substances and Herbal Preparations in Herbal Medicinal Products/Traditional Herbal Medicinal Products.

[48] Fu Y., Yang S., Jeong S.H., Kimura S., Park K. (2004). Orally fast disintegrating tablets: Developments, technologies, taste-masking and clinical studies. Crit. Rev. Ther. Drug Carr. Syst..

[49] EDQM (2025). Powder Flow. European Pharmacopoeia.

[50] EDQM (2025). Bulk Density of Powders. European Pharmacopoeia.

[51] EDQM (2025). Uniformity of Mass of Single-Dose Preparations. European Pharmacopoeia.

[52] EDQM (2025). Resistance to Crushing of Tablets. European Pharmacopoeia.

[53] EDQM (2025). Disintegration of Tablets and Capsules. European Pharmacopoeia.

[54] EDQM (2025). Friability of Uncoated Tablets. European Pharmacopoeia.

[55] EMEA (2023). Guideline on Validation of Analytical Procedures.

